# The Hospital Nursing Supplement

**Published:** 1895-10-26

**Authors:** 


"The Hospital" Nursing Mirror.

Being the Extra Nursing Supplement of "The Hospital" Newspaper.

[Contributions for this Supplement should be addressed to the Editor, The Hospital, 428, Strand, London, W.C., and should have the word "Nursing" plainly written in left-hand top corner of the envelope.]

### News from the Nursing World.

### GREAT NORTHERN HOSPITAL LADIES' ASSOCIATION.

Her Royal Highness the Duchess of York has graciously consented to become president of the Ladies' Association in connection with the Great Northern Central Hospital, and has promised a subscription of £20 towards the fund.

### GUY'S NURSES' CHORAL SOCIETY.

The Choral Society recently formed for the nursing staff of Guy's Hospital has already commenced its weekly practices. The committee consists of president, treasurer, secretary, one sister, and two staff nurses, and the society is composed of members and honorary members. Mrs. Pye Smith has accepted the office of president, and Mrs. Shaw that of treasurer, and Miss F. Nott Bower has kindly consented to be secretary to the new society, which has every prospect of a successful season.

### HOSTEL OF ST. LUKE.

The Hostel of St. Luke, in Nottingham Place, had the honour of a visit from Princess Christian and the Princess Victoria of Schleswig-Holstein on Saturday. Amongst those present to receive Her Royal Highness were: The Rev. Canon Utterton (the chairman), Dr. Champneys, Dr. F. de Haviland Hale, Mr. Pickering Pick, Dr. Roberts, Miss Vera Carter, Mr. T. H. Belfrage, Miss Phillips (matron), and several members of the committee. This hostel, established to provide surgical and medical treatment, with skilled nursing, for the clergy and their families, has proved a great boon to many patients.

### EXHIBITION OF NURSING APPLIANCES.

The Editors of *Nursing Notes* are certainly to be congratulated on the excellent exhibition of nursing appliances now open at the Trained Nurses' Club, 12, Buckingham Street. So many novelties are to be seen there that the show is of value to everybody engaged in nursing the sick, and a number of visitors have already been much interested in the various inventions of nurses, and in the exhibits of several firms who are well-known caterers for nurses' wants. The courteous explanations of the various articles displayed on the stalls, given by those in charge of them, are exceedingly helpful. The Editors of *Nursing Notes* have succeeded in enlisting a great many capable coadjutors, and they have raised general interest in this unique exhibition. It will remain open during next week, and we trust that many of our readers will take this opportunity of visiting the club; they will recognise as old friends some of the pictures of nursing subjects lent by The Hospital for the adornment of the room, and also a complete collection of books useful to nurses lent by the Scientific Press. Next week we hope to find space for an illustrated description of some of the articles shown, and in the meantime advise all our readers to make a personal inspection of the exhibition.

### WHAT ARE WOMEN WORTH?

At the National Conference of Women Workers, held this week at Nottingham, it was stated that ladies who lecture under the London County Council Technical Education Board receive £90 per annum, travelling expenses, and £5 for "any little extras." It was also announced that "clever, capable women" were required for the work. As these ladies have to board, lodge, and clothe themselves, besides providing for sickness and old age, it is probable that they will prefer serving County Councils who hold more liberal views of the remuneration due for such work, which demands a costly preliminary training, than are held by the London County Council.

### NURSING AT BIRKENHEAD WORKHOUSE.

The evidence of the medical officers at the inquiry recently held by the Workhouse Committee at Birkenhead seems to show that the nursing there leaves much to be desired. Although a number of small charges which were considered by the Board revealed individual inefficiency, the doctors apparently insisted that the system rather than the nurses themselves must be blamed for the insufficient attention given to the sick. The Guardians appeared (as reported in the local press) to consider that their having had the question of the revision of the nursing staff "under consideration" for some time, ought to have absolved them from present responsibility. This could hardly be considered consolatory by the medical officers whose work remained handicapped by the lack of an adequate nursing staff, neither could these gentlemen feel their interest in their patients' welfare much stimulated by the attitude of opposition with which their complaints were received.

### CONVALESCENT HOME AT NEW BRIGHTON.

The Convalescent Home for Women and Children occupies an excellent situation at New Brighton, and is a well-ordered and pleasant establishment. The lady superintendent, Miss Forster, is excusably proud of the pretty home-like single rooms occupied by the first-class patients, for whom a comfortable drawing-room is also provided and a large dining-room. The second-class patients have plainer sitting and mess rooms, and no single bedrooms. There is a capital recreation hall, where musical and other entertainments take place, and everything seems done to make residence at the Home agreeable. It is a good place to winter in, for the house is warmed throughout, therefore an invalided nurse could advantageously go to New Brighton at any season of the year. This prosperous-looking establishment has no fixed income, but annual subscriptions bring in about £600. Miss Forster is very anxious to add a grand piano to the recreation hall, and trusts that one may be given by some of the kind friends interested in this strictly unsectarian institution. There is accommodation for ninety-five patients, but occasionally room is made for ten extra ones when very urgent appeals for admission are received.

### DISTRICT NURSING AT STOCKPORT.

There was a large attendance at the fourth annual meeting of the Stockport Sick Nursing Association, which was held at the Court House, the Mayor presiding on the occasion. Cordial approval was given to the work already accomplished by the association, and reference was made to the increase in thank-offerings. These amounted last year to about £10, from those who had used and appreciated the services of the nurses. The handsome contribution received from the United Order of Free Gardeners and their friends was also mentioned, and the church parade which they organised, as Canon Simonds said in his sermon on that occasion, originated from gratitude for the ministrations of the nurses to one of their body. The association appears to be well framed and officered, and in all respects worthy of liberal and continuous support.

### PROMOTION FOR WARDMAIDS.

A suggestion that wardmaids might be encouraged to pick up in hospitals or poor-law infirmaries sufficient nursing knowledge to fit them to become cottage nurses, has been previously alluded to in this journal. The idea is one calculated to gladden the hearts of youthful cleaners of grates and brasses, but it may, unfortunately, raise in them hopes impossible of fulfilment. In the poor law infirmary women who do the rougher part of the ward work are certainly not those to whom the care of the sick in rural districts could advantageously be entrusted, and neither they nor the maids in hospitals are likely to meet with much encouragement from employers to go in for incidental nurse's training. Letters frequently reach our office penned by ambitious young persons who ask, in bad English and worse writing, for information as to the process by which a nurse is evolved from a wardmaid. Our correspondents' ages seem to range between seventeen and nineteen, and they have obviously met with small encouragement in the institutions where they are known, and therefore aspire to become probationers in large metropolitan hospitals. It is to be hoped that, after discovering that neither their age nor education is within measurable distance of the modern standard of nursing, they will rest contented in a sphere where they often add greatly to the comfort of the sick and the trained nurses who attend on them.

### THE RESULTS OF THE ABERDEEN BAZAAR.

The grand bazaar held at Aberdeen in aid of the District Nursing Association proved a brilliant financial success. The total expenses, including hire of rooms, lighting, advertising, music, and fitting up of stalls, only amounted to £140, and not only were the arrangements all made in a most able and economical manner, but much liberality was also shown by local tradesmen and others in sympathy with the work of the district nurses. The net proceeds of the bazaar were announced at a recent meeting of the stallholders to have reached the handsome total of £1,506; and the energetic hon. secretary, Miss Lumsden, is to be congratulated on her share in the success of an undertaking by which an admirable association has been so substantially benefited.

### "IN THIS WEEK'S 'HOSPITAL.'"

"Will you please insert the enclosed in *this* week's Hospital?" is a request often contained in letters which reach the Editor on Thursday night or Friday morning. We are glad to comply with our readers' wishes when they send us current news, but we wish they would remember that our journal is already on the bookstalls on Friday. Even without practical knowledge of printers and publishers, our readers must surely know that weekly papers are prepared *before* the date of issue. Appointments and other news should therefore be sent to the office (428, Strand) as early in the week as possible. Communications intended for "Everybody's Opinion" should be as concise as possible, and written clearly on one side of the paper only.

### BALLYMONEY WORKHOUSE INFIRMARY.

The Ballymoney Guardians have been influenced by a communication from the Local Government Board to reconsider their refusal to appoint a night nurse for their infirmary. A large majority voted in favour of rescinding the former resolution, but according to the local press thirteen guardians afterwards voted that a "partially trained" night nurse should be secured.

### PREVENTION OF CRUELTY TO CHILDREN.

The Society for the Prevention of Cruelty to Children has a noble list of patrons, headed by her Majesty the Queen, whose approval of the society should secure for it world-wide support. In the words of the Secretary: "It does not remove children from wretched homes, nor relieve parents of their responsibilities, but seeks to improve homes and to enforce responsibilities." The director and secretary, the Rev. B. Waugh, will gladly receive subscriptions at 7, Harpur Street, London, W.C.; or they can be sent to the hon. treasurer, the Hon. Evelyn Hubbard, at the same address.

### SHORT ITEMS.

The Bashford Guardians have agreed to subscribe to local district nursing associations, being convinced that "the recovery of the sick is much expedited by good nursing."—The Hull District Nursing Association is sorely in need of help, its financial condition having necessitated a reduction of the staff at a season when the nurses' services are specially in demand.—A concert in aid of the Bainbridge Nursing Society was recently held in the Town Hall, when an excellent programme was thoroughly appreciated by a large audience.—At the Torquay County Court the Torquay Nurses' Institution recovered a debt of £9 for services rendered in nursing, the defendant admitting the debt and promising to pay costs.—The inquiry into nursing matters at Bangor Workhouse appears to show urgent need for the reorganisation of that department.—We learn with regret that Miss Pattison's health has necessitated her resignation of the matronship of Lewisham Infirmary.—The medical officer of Mount Mellick Workhouse has recommended that a night nurse be appointed for the infirmary, and a paid attendant for each of the imbecile wards.—The Woman Inspector whom the St. Pancras Vestry have decided to secure will probably begin work with the new year, and her duties will lie mainly amongst the laundries, workshops and workrooms where women and girls are employed.

### Elementary Physiology for Nurses.

By C. F. Marshall, late Surgical Registrar Hospital for Sick Children, Great Ormond Street.

### XII.—THE NERVOUS SYSTEM.

The nervous system consists of two main parts, the central and peripheral, the former comprising the brain and spinal cord, the latter the nerves going to all parts of the body. The general purpose of the nervous system is to bring all parts of the body into relation with one another, and to enable the parts to work in harmony.

When examined microscopically the nervous system is seen to consist of two kinds of elements, nerve cells and nerve fibres. The nerve cells have branching processes; the nerve fibres do not branch. We may compare the nerve cells to an electric battery, which generates an impulse, and the nerve fibres to the wires, which convey the impulse.

The central nervous system develops very early, and is really part of the skin. At an early stage in the development of the embryo a groove appears, which becomes gradually closed in above to form a hollow tube along the back of the embryo. This tube forms the brain and spinal cord.

*The Spinal Cord* is oval in section. At its upper end it passes gradually into the brain; its lower end terminates in a tapering point. The substance of the spinal cord consists of two kinds of matter, called the grey and white matters. The grey matter forms the more central part, and contains the nerve cells; the white matter forms the outer portion, and consists mainly of nerve fibres.

*The Brain* consists of (1) the cerebral hemispheres, which are of large size with a convoluted surface; (2) the cerebellum, or small brain, situated underneath the former; (3) the medulla oblongata, which is continuous with the spinal cord. The ventricles of the brain are spaces continuous with the central canal which runs down the spinal cord.

The spinal nerves, of which there are 31 pairs, arise from the spinal cord, and supply all parts of the body. They pass out through holes between the vertebræ. Each spinal nerve arises by two roots—a posterior and an anterior. These separate roots unite together before leaving the vertebral column. The nerve fibres are distributed—some to muscles, some to the skin, some to glands.

### Functions of the Spinal Nerves.

(1) Irritation of a spinal nerve, for instance, one supplying the arm, causes twitching of the muscles to which it sends branches, and also pain referred to the nerve endings in the skin of the part supplied by it. (2) Irritation of the posterior root of the nerve causes pain only. (3) Irritation of the anterior root causes contraction of the muscles only. Hence the distinction between motor nerves and sensory nerves, which are separate in the two roots of each spinal nerve, but combined in the main trunk of the nerve, the posterior root consisting of sensory nerves, the anterior of motor ones. (4) Section of a spinal nerve causes both motor and sensory paralysis. The nerve no longer has any power over the muscles supplied by it, and sensation of pain is lost. (5) Irritation of the distal (or further) end of the cut spinal nerve causes the muscles to contract. (6) Irritation of the proximal end (the end nearest the brain) causes pain. This pain is referred to the part of the skin to which the nerve is distributed. Thus, after amputation of a leg, the patient will often imagine he feels pain in his toes, owing to some irritation of the end of the nerve in the stump. The nerve formerly gave sensation to the toes, and the message sent to the brain is still referred to the toes, owing to former habit. (7) Section of the posterior root of a spinal nerve causes sensory paralysis, or loss of sensation in the parts supplied by it. (8) Irritation of the distal end of this root causes nothing; but irritation of its proximal end causes pain, because the fibres are all sensory ones, passing upwards towards the spinal cord and brain. (9) Section of the anterior root causes paralysis of the muscles supplied by the nerve. (10) Irritation of the distal end causes movements of these muscles; irritation of the proximal end causes nothing, because the fibres in this root all pass outwards from the spinal cord to the muscles. Hence a motor nerve is one, irritation of which causes contraction of muscles, provided it be in connection with them. A sensory nerve is one, irritation of which causes pain, provided it be in connection with the spinal cord and brain, even though it be severed from the part in which the pain is felt.

### Nursing in Rome.

### IV.—THE CHILDREN'S HOSPITAL.

This was formerly a monastery, the one to which Tasso retired and in which he died. Its position commands a magnificent view of Rome and the Campagna.

It first became a children's hospital through a certain Duchess Salviati, who brought a few children here to be cared for, and since that time it has grown until now there are a hundred and fifteen little ones all well cared for by the good Sisters of St. Vincent de Paul. This is not a free hospital—every child must be paid for; it is made use of by the rich as well as the poor owing to its high character for surgical and nursing skill. Those who are well off pay two francs a day for each child, while for the very poor the municipality pay one franc eighty centimes each patient. No matter what the social condition of the child, all receive exactly the same treatment.

The cost of keeping up this hospital is about 70,000 francs a year (£2,800). This is not too much, as the children have the best of everything without stint; 2,000 francs a month are paid for milk, 500 francs for white bread and more for brown, 800 francs for wine, and 500 francs every three months for medicines, and a large sum for bandages.

There is an isolated ward for infectious cases, although no such are knowingly taken in. The majority of the patients brought here are suffering from diseases of the limbs; the number of successful operations performed by celebrated surgeons within this hospital is very large.

There is a small operating-room with ward attached and an excellent theatre with everything needful, all in excellent order. In the three large wards for boys the snowy cots are ranged on either side on scrupulously clean brick floors. Each cot has a convenient wooden bed-tray across it for food or toys. The girls' side is in every respect as good as the boys'. Many had dolls in bed with them, and such as were able were trotting about. The whole place was redolent of sunshine and warmth, and we were glad to see reed curtains hung about to keep the flies from tormenting the little sufferers.

The kitchen is large and convenient, with a trap window through which the food is passed; it contains an excellent range capable of cooking a great deal at a small expenditure of fuel. The food is very good, but it is piteous to see the wee white faces turning from it because they have no appetite. A slate is hung on each bed, bearing the name and the disease of the occupant.

The Sisters take it in turn to keep watch at night, and one and all of these noble women are unceasing in their care.

The old cloisters round the hospital are very interesting, so also is the church, with its beautiful rich frescoes, but more attractive to us were the tiny sufferers and the good women who spend their lives in trying to alleviate their pain.

### On Urine Testing.

### I.—INTRODUCTORY.

Anything like complete analysis of urine, or, indeed, any attempt to interpret the meaning of abnormal reactions, is out of the province of the nurse; but the observation and recording of variations in this secretion is peculiarly one of her functions, for it will always be the case in private practice that the nurse, who is constantly on the spot, must act as the clinical clerk to the practitioner, whose visits take place at more or less lengthened intervals. Just as the nurse records the pulse, the temperature, and the respiration, so must she be able to record the conditions of the excreta. Especially is this the case in regard to the urine, a secretion which alters greatly in character from day to day and from hour to hour.

It need hardly be said, however, that so far as any investigations beyond mere inspection may be found necessary, it will be well that they be made with as little parade and publicity as possible. To the clean no doubt all things are clean, but the public do not always understand that view of the matter, and it is as well not to try too severely their appreciation of the scientific nicety of many disagreeable processes.

The chief points on which information may be required are: colour; opacity; character of sediment, if any; total quantity in 24 hours; specific gravity; presence of albumin; and presence of sugar and its amount. In some cases the quantity of urea.

The principal abnormalities as to colour are: (1) great paleness, which may occur in some forms of kidney disease, in diabetes, and in hysteria; (2) increased depth of colour, such as often occurs in febrile complaints; (3) "smokiness," a colour somewhat as if a little soot had been added, which is generally an indication of an admixture of blood, but is seen also in cases of carbolic acid poisoning; and (4) darkness from the presence of bile, ranging from a deep yellow tint almost to blackness.

In regard to opacity, it is to be noted whether it is present at the moment the urine is passed, or only developes in cooling.

The urine sometimes contains the salts of uric acid (called urates or lithates) in so concentrated a form that, although they remain dissolved so long as the urine is warm, they are cast out of solution so soon as this is cool. This leads to the staining of the vessel by the deposit of these salts upon it, the process being analogous to that by which, when a hot concentrated syrup is allowed to cool, crystals of sugar-candy are deposited on the sides of the pan or on strings suspended in it. In either case the addition of water and the re-application of heat causes the deposit to dissolve, and thus it is that an opacity occurring in urine on cooling, which disappears on the application of heat, is usually caused by urates.

For examining urine test tubes are employed, and it is usual to apply heat by means of a spirit lamp. It is worth noting, however, that a saucepan full of boiling water is a very convenient mode of obtaining heat for the purpose. The pan may be removed from the fire and placed near the window or the light, and the test tube held in the hot water, when its contents will soon be brought to the required temperature.

After urine has been standing for a time certain changes take place, by which the uric acid is set free from the urates, and this uric acid, being much less soluble than the salts, is cast down in the form of crystals, which often attain such a size as to be clearly visible. These form a deposit of red sand, somewhat like grains of cayenne pepper. When once formed, this deposit does not re-dissolve on the application of heat as the salts do. These crystals do ot usually adhere to the sides, but roll about like a clean sand on the bottom of the vessel.

Sometimes urine is thick from the presence of pus blood, mucus or bacteria. Such opacity does not dissolve on the application of heat, and it may be said at once that the discovery of any opacity which does not at once clear up on the application of heat should lead the nurse to preserve a specimen for the doctor to submit to further examination. A word here as to the preservation of specimens. The strictest cleanliness is necessary, or fermentation will rapidly take place, and the specimen will be spoiled. The vessel, therefore, into which the urine is passed, as also that in which it is to be preserved, should be scalded with boiling water. It not infrequently happens that a medical man wishes samples to be sent to him occasionally for more complete examination. In the country, unless a special messenger is employed, the best way of doing this is by Parcels Post, but whether in town or country, the sample should be properly packed. There are few things so calculated to enrage a doctor as the delivery at his kitchen door of a specimen of this sort roughly twisted up in a piece of paper.

A four-ounce bottle is usually large enough to hold a sample; it should be first well scalded, then should be filled to the bottom of the neck, and be well corked (but air should be left in the neck, otherwise the bottle will be very liable to burst in transit), and then be labelled with name and date and time of day. It should then be wrapped in several thicknesses of paper and packed in a wooden or tin box. A tin canister, such as mustard, biscuits, coffee, &c., are sold in, is the best. The parcel should be carefully directed, and above the direction should be written "Professional—Private." In this way no unseemly accident will be likely to occur.

There are many cases in which it is desirable to ascertain whether or no albumin is present in the urine. It is not suggested that any examination made by the nurse is to take the place of that instituted by the doctor, but there are times —as, for example, during convalescence from scarlet fever and during the chronic stages of Bright's disease—when it is important that a morning and evening examination should be made, while medical visits may be few and far between. A nurse, therefore should be able to ascertain the presence of albumin, and to judge roughly as to its amount.

### Canine Collectors for Hospital Funds.

From 1890 to 1895 the treasury of the Bristol Royal Infirmary has been benefited to the extent of about £33 by the sagacious collections of a lively Irish terrier named "Punch," who is entitled to have his services chronicled, for there are many human beings who have done less than he in the sacred cause of charity. In the course of six years his average annual takings may be reckoned at £5 10s.; the last, that of 1895, consisted of 2,600 coins of all sorts. There is a large tin case on a table in a hostelry in Bristol which is his savings box, and in this he deposits whatever coins are given him. His antics and gambols are amusing enough; he will sit about attracting your attention and then look up at you with solicitous eyes, touching your pocket with his paw. The infirmary must be congratulated upon having such an efficient coadjutor. If this plan were extensively followed the Hospital Funds of England would be appreciably augmented, and nobody feel a penny the worse. "Punch" is the proud possessor of two silver medals bestowed upon him by the hospital, and a handsome inscribed silver collar.

### London Nurses' Training Schools.

### LECTURES TO PROBATIONERS.

The courses of lectures delivered to the nursing staffs of the various training schools are, in most cases, already arranged, and as they are of interest to so many of our readers, we propose to devote space for some of the syllabuses, These we shall be glad to receive from all nurse-training schools—metropolitan, provincial, and colonial.

### THE NIGHTINGALE TRAINING SCHOOL—ST. THOMAS'S HOSPITAL.

Present Season.

October, 1895: Dr. Sharkey's lectures to the nurses commenced on October 7th, but we have not yet recived the syllabus.

Dr. Sharkey's Syllabus of Lectures for October, 1894, was as follows: October 4th, "On the Anatomy of Cells." October 11th, "On the Functions of Cells." October 18th, "On the Composition of Blood." October 25th, "On the Circulation of the Blood." November 1st, "On Diseases of the Heart." November 10th, "On the Classification of Foods." November 15th, "On the Digestion of Food." November 22nd, continued. November 29th, "On the Excretory Organs." December 8th, continued. December 13th, "On Inflammation." A written examination on the lectures is held in December.

Classes for Nightingale Probationers.

All through the year the Home Sister holds classes for the probationers on Monday, Tuesday, Wednesday, Thursday, and Friday, from eleven a.m. to twelve, giving instruction on elementary physiology, anatomy, hygiene, medical terms, abbreviations, reading of bed tickets, weights, measures, &c. A limited number of probationers attend each class, so as not to take too many away at once from the wards.

During September, the Home Sister held bandaging classes three times a week, from eleven a.m. to half-past twelve.

### LONDON HOSPITAL TRAINING SCHOOL FOR NURSES.

Nursing lectures are given every Wednesday at eight p.m. The first course on "The General Details of Nursing," by the Matron, Miss Lückes, commenced on September 4th. The second course, on "Elementary Anatomy and Surgical Nursing," by Mr. C. W. Mansell Moullin, F.R.C.S., surgeon to the hospital, begins early in November. A limited number of ladies are admitted on payment of half-a-guinea for each course.

Preliminary Training at Tredegar House.—Lectures on elementary physiology and anatomy are given to the pupil-probationers in the clinical theatre by Dr. Sequiera and Dr. Dawson. Lectures on elementary hygiene are given by Dr. Schorstein and Dr. Hadley. Examinations are held on these subjects at the conclusion of each course, after which the pupil-probationers are transferred to the hospital. Before coming into the hospital pupil-probationers are also examined in bandaging, practical bed-making, and sick-room cookery.

### MIDDLESEX HOSPITAL.

Three courses of lectures are delivered annually to the nurses and probationers of this hospital on "Anatomy," by Mr. A. Pearce Gould; on "Physiology," by Dr. Coupland; and on "General Nursing," by Mr. E. A. Fardon. The date for beginning the lectures of the coming season is not yet determined.

### GUY'S HOSPITAL NURSES' TRAINING SCHOOL.

The lectures on nursing are given during the months of September, October, November. The first four by the Matron on Nursing Ethics, the remainder by Miss Oxford (sister of Phillip Ward) on Practical Nursing.

During the months of December, January, and February, the lady pupils and staff nurses are taught the elements of pharmacy and dispensing, in the dispensary of the hospital by Mr. Collier, the head dispenser. The course includes a series of lessons upon the sources, properties, and uses of various drugs, with practical instruction in the preparation of mixtures, pills, and powders.

After each course of lectures an examination is held, and those candidates who fail to satisfy the examiners are ineligible for promotion for a year, and if unsuccessful at the next examination have to leave.

### ST. MARY'S HOSPITAL.

Lectures to Nurses, 1895-96, Commencing Thursday, October 3rd, 1895, and Continuing on succeeding Thursdays at Five p.m.

Syllabus of Lectures on Elementary Anatomy and Physiology, and on Surgical Nursing, by A. Q. Silcock, B.S., M.D., F.R.C.S.Eng., Surgeon to Out-patients, St. Mary's Hospital, Surgeon Royal London Ophthalmic Hospital: Lecture 1, "Elementary Anatomy and Physiology." Lecture 2, "Elementary Anatomy and Physiology." Lecture 3, "Healing of Wounds—Inflammation." Lecture 4, "Treatment of Wounds—Antiseptic Surgery." Lecture 5, "Surgical Infective Diseases—Erysipelas, Cellulitis, Septicæmia, Pyæmia, and Tetanus." Lecture 6, "Fractures, Simple and Compound." Lecture 7, "Treatment of Fracture—Mode of Repair—Splinting and Bandaging." Lecture 8, "The Surgical Ward—The Sick Room." Lecture 9, "Surgery of the Head and Spine." Lecture 10, "Surgery of the Chest and Abdomen." Lecture 11, "Surgical Emergencies—Shock, Hæmorrhage, &c." Lecture 12, "Bed Sores—Burns—Ulcers."

Syllabus of Lectures on the Elements of Nursing, by A. P. Luff, M.D.Lond., B.Sc., M.R.C.P., Physician to Out-patients, St. Mary's Hospital, Lecturer on Forensic Medicine, St. Mary's Medical School: Lecture 1, "The Digestive System." Lecture 2, "Diseases of the Digestive System." Lecture 3, "Food in Health." Lecture 4, "Food in Disease." Lecture 5, "The Circulatory System." Lecture 6, "Diseases of the Circulatory System." Lecture 7, "The Respiratory System." Lecture 8, "Diseases of the Respiratory System." Lecture 9, "The Urinary System, and Examination of Urine." Lecture 10, "Fevers." Lecture 11, "Fevers." Lecture 12, "Treatment of Fevers—Arrangements and Hygiene of the Sick Room—Ventilation—Temperature—Disinfection."

Syllabus of Lectures on Obstetric Nursing, by Montagu Handfield-Jones, M.D.Lond., Obstetric Physician to St. Mary's Hospital, Joint Lecturer on Midwifery and Diseases of Women to St. Mary's Medical School, Physician to the British Lying-in Hospital: Lecture 1, "The Normal Position and Relations of the Pelvic Viscera—Displacement of Organs—The Douche—Its Mode of Use and Therapeutical Action." Lecture 2, "Menstruation—Its Abnormalities." Lecture 3, "Pelvic Inflammation—Its Course and Some Points in the Treatment—Sequelæ to be Guarded Against—And Precautions Necessary." Lecture 4, "Pelvic Inflammation—Abscess of the Pelvis—Application of Leeches." Lecture 5, "Ovarian Tumours—Their Origin and Mode of Growth—The Operation for Removal." Lecture 6, "Ovariotomy—The Preparation of the Room and Care of Instruments—After Treatment—Abdominal Drainage."

### CHARING CROSS HOSPITAL.

There are three sets of lectures given to the nurses at this hospital, twelve in each course. The first, consisting of Dr. Willcock's lectures on "Anatomy and Physiology," commenced on the first Monday in October.

(*To be continued.*)

### Royal British Nurses' Association.

### QUARTERLY MEETING OF THE GENERAL COUNCIL.

Admittance to the council meeting on Friday, 18th inst., having been refused to the representatives of the Press, we are unable to give a verbatim report of the proceedings. We are able, however, to give the following report, which we believe fairly records the gist of the proceedings.

About eighty persons were present, including Sir James Crichton Browne (in the chair), Sir Spencer Wells, Bart., Mr. Pickering Pick, Dr. Alexander Davey, Mr. Grant, Dr. Bedford Fenwick, Miss Thorold, Miss Mollet, and others. Her Royal Highness Princess Christian, president of the association, was not at the meeting.

The minutes of the previous meeting were read and confirmed.

It was then proposed by Dr. Bezley Thorne, and seconded by Mr. Brudenell Carter, that the following report of the Executive Committee should be adopted:—

The Executive Committee have to report that ten nurses were registered by the July meeting of the board, and that thirteen have been elected members of the corporation; another ten have withdrawn, and four have died. The hon. secretary of the Scottish branch also reports the registration and election as member of one nurse. The Executive Committee regret to report that the treasurer and the medical honorary secretary have intimated their intention to resign. For the first-named office they have the pleasure to recommend the appointment of Mr. Langton, who has kindly consented to serve. At the meeting of the Registration Board, held on July 26th, it was decided to recommend the omission from Regulation I. of the concluding sentence: "The Registration Board has the power to waive the latter condition under exceptional circumstances, in so far as it relates to the number of beds"; and that the regulation should run as follows: "Applicants for registration must produce proof that they are of good character, and that they have been engaged in work for three years in hospitals or infirmaries containing at least forty beds, of which time not less than twelve months must have been spent in a general hospital." That recommendation was unanimously adopted by the Executive Committee, which met in ordinary meeting on the 4th inst. It has been definitely decided that the initial meeting, preparatory to the formation of a local centre, will be held in the library of the Sussex County Hospital, Brighton, on November 7th, at five p.m., thanks to the exertions of the lady superintendent, Miss Scott. Miss East, lady superintendent of the National Hospital, Queen Square, London, is actively engaged in endeavouring to initiate a similar meeting at Newcastle-on-Tyne. Miss Mollett, of Southampton, whilst warmly supporting the scheme, regrets her inability to take active measures towards its realisation until early in next year. On reconsideration, the Executive Committee recommend the General Council to rescind their resolution of July, and empower the Executive Committee to arrange for a further course of educational lectures.

Dr. Bedford Fenwick, in speaking of the proposed rewording of the regulations for registration, appears to have objected to nurses trained in country institutions with less than forty beds being rendered ineligible. A discussion, in the course of which it was, we believe, remarked by a member that nurses with only cottage hospital training appeared to be at present admissible, ended in a postponement of this matter.

### Treasurer's Report.

The financial history of the association during the last three months has been uneventful—during this period of the year our expenditure goes on as usual, and our receipts are small. However, I am glad to report one very gratifying circumstance—that a medical member of the association, who desires his name to be withheld—has presented us with £100. Some years ago, in the old offices at Oxford Circus Avenue, the association laid down some linoleum, and in consequence, at the end of our tenancy, the flooring was found to be in a condition of dry rot, and we have been compelled to pay about £20 to make good the damage. We have recently issued another whip to members who have not yet paid their annual subscriptions. About £92 is owing from this source. May I remind the council that the annual subscriptions for three years are becoming due?

It was proposed by Mr. Grant, and seconded by Dr. Davey, and agreed, that the treasurer's report should be accepted. From the remarks made by Dr. Bedford Fenwick, it did not appear that the present financial condition of the association was particularly prosperous.

On the motion of Mr. Pickering Pick, the resignation of Dr. Bezley Thorne was accepted. The Chairman proposed a vote of thanks to Dr. Thorne for his valuable services during the four years in which he had held office as medical honorary secretary to the association. This was seconded by Miss Meyrick, and carried.

Dr. Calvert's resignation of the post of honorary treasurer was accepted, and very hearty thanks were tendered to him for his past services on the motion of Dr. Bedford Fenwick, seconded by Miss Okell.

The office of honorary treasurer, vacant by the resignation of Dr. Calvert, was conferred upon Mr. Fardon, whilst Mr. Langton succeeded Dr. Bezley Thorne as medical honorary secretary.

Dr. Calvert warmly expressed his gratitude for the able assistance which he had received from the secretary, Miss Ravenhill.

In laying down the office of medical honorary secretary, Dr. Bezley Thorne made a statement of considerable importance. His well known loyalty and indefatigable zeal in the interests of the association are so universally understood that it is obvious that only a strong sense of duty could have forced him to take so serious a view of its present position, Referring to the internal dissensions, of which not only the members of the Royal British Nurses' Association but also the general public have long been aware, Dr. Thorne spoke of the hindrance which a small and occult organisation had for years raised within the association. That organisation had endeavoured to make it difficult or even impossible for all such officers as were not prepared to be obedient to its policy to remain at their posts.

We understand that this policy appeared to be one that would nullify the influence of those medical members, matrons, and nurses who, according to the constitution of the association, regarded the vocation of nursing as occupying a subordinate position in attendance on the sick. The policy seemed also to include the elimination from its committees and officials of all who were not prepared to yield to irresponsible influence and dictation. It was further stated that the medical profession would never agree to recognise an organised body of nurses who aimed at being independent, like the clique in question, of medical control.

### Minor Appointments.

Government Civil Hospital, Hong Kong.—Miss Mary Elizabeth Mead, late Sister Blizard at the London Hospital, has been appointed Sister at the Government Civil Hospital, Hong Kong, and sailed on October 6th, 1895. Miss Mead holds the diploma of the London Obstetric Society, and we wish her all success at the hospital where so many Londoners have already done excellent work.

### Where to Go.

East End Mothers' Home.—The opening of the new buildings, 306, Commercial Road, London, will take place on November 12th, at quarter-past three. The ceremony will be performed by the Lord Mayor.

### Everybody's Opinion.

[Correspondence on all subjects is invited, but we cannot in any way be responsible for the opinions expressed by our correspondents. No communications can be entertained if the name and address of the correspondent is not given, or unless one side of the paper only be written on.]

### TEACHER AND LECTURERS.

"E. L. H." writes: I enclose copy of letter sent to the *Nursing Record* respecting some statements made in respect of professional women, &c: "Madam,—I wish to state, as a National Health Society's student, trained at the Chelsea Infirmary by Miss De Pledge, for teaching the poor to make the best use of materials at hand in nursing in their own homes, that I stayed in the infirmary for one year and received careful attention, gaining great experience whilst there. No one can be received for less than six months, but students may remain longer if they wish to. The National Health Society's students are not qualified to nurse, nor do they pretend to be trained nurses, where questions of life and death are involved; but as teachers of the best methods of helping the poor to make the best use of the materials at their disposal in their own homes.—Yours truly, TRUTH."

### THE LIVES OF ASYLUM WORKERS.

"AN OLD NURSE" writes: The letters in THE HOSPITAL concerning "Asylum Nurses" have interested many besides myself, and I would like to congratulate the writer of the first one on her success in arousing a doctor to take the defensive side. Medical men are very ignorant concerning our every-day lives, and restless, sleepless nights spent in or near the refractory wards, after which we are expected to be on duty at six a.m., while the doctors sleep on peacefully in a private wing. Our breakfast at seven a.m. consists of cold tea or coffee, burned bacon, and brown bread and margarine, and we are expected to work on the strength of that meal till about one o'clock, when our hunger is satisfied with half-boiled vegetables, raw meat, and burned pudding. About five or six p.m. another scanty meal is provided, *i.e.*, tea and bread and butter, whilst the medical staff are provided with a three-course dinner, and how many more meals they have I am not aware. We have to work fourteen and sixteen hours (as the case may be) on three meals a day. We are often asked to divide our half-hour for meals with another nurse, and have to swallow our food in ten minutes. We are dragged by violent patients in the recreation courts; we have to stand the summer's heat and the winter's snow; we are kicked and our hair is pulled, whilst we have to answer for a patient's bruises, when the blood is running down our own cheeks. In fact, it is "kill the nurses if you like, but be careful not to bruise the patients." We are reckoned by outsiders as a lazy class and not of the strictest character, and after once being an asylum nurse it is not easy to find another post, for nobody seems to wish to have you. I would not for a moment say that we are perfect, as we have many temptations, and many young women of good character, refinement, and education have to suffer through the behaviour of the minority. At any rate, it is time that an irritating and unreasonable prejudice against asylum workers should be swept away. We do work which the public does not discover. We have to listen to profane language, and come into contact with the lowest of moral characters, with danger to our souls as well as our bodies. Whatever religious denomination we may belong to, we are expected to sacrifice it at the Church of England altars. If any reader of THE HOSPITAL is anxious to become an asylum nurse, let me as an experienced one warn her that she must be prepared to sacrifice all human feelings, to obey many masters, to risk indigestion from badly-cooked food, starve through not eating, or become anæmic from want of nourishment. If she is a Christian, I would advise her to leave her religion outside the idiosyncrasy of the asylum walls. My own experience has been gained in two of the leading English asylums. Perhaps things are better in "Bonnie Scotland."

### A TRAINED MIDWIFE WANTED.

"A CHESHIRE NURSE" writes: May I make known through your columns a great want we have in this parish? Any one willing to supply this "want" will not find the process lucrative, but there is good work to be done by a certificated midwife who would come and save us from the company of women who call themselves "midwives." In addition to the five shillings which they charge for their services they require drink, "to the full three shillings worth of sperrits, mum—aye, and sauce me well if it ain't there fur 'em," has been said by several of the "mothers" in the district. They leave the case altogether at the end of five days; and only last week a district nurse visited a case which had been attended by a neighbour, whose only qualification was that she "had had ten of her own," and so "ought to know som'ut about it, nurse." The effect of such knowledge was that the mother was found, thirty-six hours after delivery, without even her face having been washed, and a temperature of 102 degrees. The poor babies are fed, directly after their first wash, upon "gruel," made by pouring boiling water upon oatmeal, which is then made very sweet. The patient is invariably expected to get out of bed with bare feet (all the floors of the cottages here are of stone), and sit in a chair while the bed is changed, some six or eight hours after she has been confined, to suit the daily occupation of the woman attending the case. In addition to this, the conversation and behaviour of these so-called "midwives" is indecent and revolting in the extreme. Now, we want a trained lady who, for love of the work and for the sake of security, comfort, and safety to many poor mothers, would try and work some reforms. She must have some means of her own; the workmen receive such small wages—15s. or 16s. a week being considered "good"—that little help can be expected from them; but I feel sure a lady with a kind heart and plenty of tact would soon accomplish great things. A well-worked Maternity Club would do wonders. Any lady would receive a most kindly welcome from Rector and doctors. The parish nurse, who is not allowed to attend monthly cases, would be willing to share her charming little cottage, and board and lodge such a worker for about 10s. a week.

### WORK IN ASYLUMS.

"NURSE K." writes: I have read with great interest the letters from asylum nurses in the three last numbers of THE HOSPITAL. I have found both hospital and asylum experience hard, but hospital is preferable to an asylum (taking it for granted that the nurse wishes to do her duty thoroughly). In hospital she is treated with respect by officers and patients, and encouragement is given her daily by patients who are recovering in relieving their sufferings. Nurses have their time off duty regularly each day, and their meals are comfortably prepared for them. Asylum nurses have insults and blows for which the patients are not, of course, to be held responsible; but the officers often treat nurses as necessary evils, especially in private asylums. The work is very laborious, for it includes scrubbing, cleaning grates, windows, and knives, washing greasy plates and dishes, and shaking carpets. The preparation for meals, and the feeding of those incapable of helping themselves, and many other duties keep a nurse from sitting down much. The hours are very long, and in one of the leading mental asylums only one meal a day did the nurse get in peace out of the ward. In applying for a post as mental nurse some time back I mentioned that I had the Psychological certificate, but was told by the doctor that he thought no more of nurses for their gaining that. This seems to be the kind of encouragement usually given in mental work. I think there is much room for improvement in the treatment of mental nurses. Why should they be thought less of than sick nurses?

EAST DULWICH INFIRMARY.

We, the undersigned nurses of the East Dulwich Infirmary, beg to contradict the utter falsehood contained in the letter of a "Working Nurse" which appeared in The Hospital of October 19th, and we hereby wish to express our entire satisfaction both with the quality and quantity of the food provided for us by the authorities:—

L. Legg, A. Mower, H. M. Porter, P. F. Chatfield, A. B. Curtis, K. Gilbert, C. J. Morgan, M. Adams, E. Sample, R. Stanley, A. W. de Chastelain, G. Hopkins, E. R. Turner, H. F. Keeping, L. E. Stickland, M. J. Bayley, T. Davy, H. M. Orrell, E. Fenemore, E. N. Childe, L. Thurling, M. Davies, E. Smith, L. de Chastelain, M. Jones, N. Durtmall, G. Chapman, L. Hunt, A. E. Madell, R. Eastman, G. Humphreys, A. Walkerley, M. Thomson, L. Tice, E. A. Kidson, E. Bracewell, F. Ruffell, F. E. Latham, S. A. Milne, E. S. Taylor, C. Winifred Jones, N. M. Dibble, E. Robarts, M. Rogers, F. Cooper, M. Williams, M. C. J. Jenner, E. Bradnam, S. Ward, H. M. Cannwack, M. Briscoe, H. Skinner, E. M. Coote, L. S. H. S. Arnold, L. M. Whiteside, A. M. Meadows, F. Mocock, J. Owens, F. E. Power, E. M. Harding, and E. Worseldine.

"A Probationer" writes: Some people tell falsehoods for fun, some because they cannot help it, while others do it out of a malicious spirit. With the last-mentioned we must class a correspondent in The Hospital who terms herself "A Working Nurse." I hope you will at once allow me to contradict her statements that the food provided for the nurses at this infirmary is not both liberal and good.

"One of the Nurses" writes from St. Saviour's Infirmary, East Dulwich: Will you be good enough to repudiate the erroneous statement which appeared in The Hospital of the 18th concerning the food supplied to the nursing staff of this infirmary? It is good, wholesome, and well cooked, and far superior to that given in most hospitals.

"E. S. T." writes: In The Hospital of last week I saw a letter from "A Working Nurse," speaking about what she termed "the bad feeding" at the East Dulwich Infirmary. Being myself a nurse at that institution, I could not think of allowing such a false statement to go before the public without doing what lies in my power to remedy such a misleading account. I have now been here over three years, and have never yet had occasion to find fault with the quantity or quality of our food, least of all now, when it is better than ever it has been. Great was the indignation of the nurses all over the infirmary on reading "A Working Nurse's" letter in your issue of last week, and they agree with me in saying that such a statement is utterly false.

NURSES' DIET IN INFIRMARIES.

"A Private Nurse" writes: As I once nursed for three years in an infirmary I cannot let the remark on the question of diets pass without saying that since those days I have been in several hospitals in London, taking temporary duties, but never has the food excelled, nor the table been so well provided, as that for nurses at the Lambeth Infirmary. There the nurses' meals were looked after by the matron, Miss Griffiths, and certainly did her great credit, and earned for her gratitude from us all.

[We have great pleasure in inserting these letters, and congratulate the writers on their loyalty to their institution. The letter of the "Working Nurse" has served a useful purpose in bringing out such abundant proof of the good catering at Dulwich Infirmary. We regret that we cannot make space this week for several other letters on this subject.—Ed. *T. H.*]

### Royal British Nurses' Association.

A meeting with a view to the formation of a local centre was held at Newcastle-on-Tyne, on Tuesday, 14th inst., of which details will be published in the November number of the "Nurses' Journal."

### Appointments.

Farnworth and District Infectious Diseases Hospital.—Miss Theresa Sherwood has been made Matron of this hospital. She received her training at Salford Union Infirmary, and was head nurse at Darlington Fever Hospital. We congratulate her on her appointment.

Corbet Hospital, Stourbridge.—Miss Kate E. Richards has been appointed Matron of this hospital. She was trained, and afterwards held the post of staff nurse and sister, at the General Hospital, Birmingham, where she had charge of wards for three years. Miss Richards was matron at Berkeley Hospital for a year and a half, and held a similar post at Market Drayton for over three years. Many good wishes accompany Miss Richards to her new work, and her testimonials are excellent.

### Notes and Queries.

The contents of the Editor's Letter-box have now reached such unwieldy proportions that it has become necessary to establish a hard and fast rule regarding Answers to Correspondents. In future, all questions requiring replies will continue to be answered in this column without any fee. If an answer is required by letter, a fee of half-a-crown must be enclosed with the note containing the enquiry. We are always pleased to help our numerous correspondents to the fullest extent, and we can trust them to sympathise in the overwhelming amount of writing which makes the new rules a necessity. Every communication must be accompanied by the writer's name and address, otherwise it will receive no attention.

### Queries.

(13) *Male Attendant.*—Please tell me names of asylums where there are lectures to prepare male attendants for the Medico Pyschological Examinations?—*Attendant.*

(14) *Midwife.*—Is a midwife who has passed the examination of the London Obstetric Society entitled to call herself a Licentiate and put letters after her name? I wish to thank you for the very valuable assistance given to nurses by the articles in The Hospital.—*Missionary Pupil.*

(15) *Male Nurse.*—Where can I get trained as male nurse?—*Enquirer.*

(16) *Syrup.*—I should be glad to know if Mother Seigel's Syrup is really harmless, and what it contains?—*District Nurse.*

(17) *Infection.*—Will you tell me, please, whether you consider it safe for an infirmary and an infectious hospital to employ the same chaplain?—*Infirmary Nurse.*

### Answers.

(13) *Male Attendant (Attendant).*—You had better write to the hon. secretary of the Association, Dr. Spence, Burntwood Asylum, Lichfield.

(14) *Midwife (Missionary Pupil).*—Thanks for kind appreciation of our efforts to help nurses. A nurse who has passed the examination you ask about can only call herself *Diplomée*, not Licentiate.

(15) *Male Nurse (Enquirer).*—See answer No. 12 in The Hospital of October 19th.

(16) *Syrup (District Nurse).*—In a pamphlet which contains papers on patent medicines, reprinted from *Hygiene*, there is a letter from Mr. Alf. W. Stokes, F.C.S., F.I.C., public analyst, in which he says of Mother Seigel's Syrup: "I find it to be a complex mixture containing treacle, borax, aloes, capsicum, and liquorice. The active ingredient is aloes, of which I extracted from the 4 oz. mixture 120 grains."

(17) *Infection (Infirmary Nurse).*—This depends largely on the chaplain, on the hygienic condition of the isolation hospital, and on the nature of the diseases treated there. We have found that the risk of infection being "carried" rests largely on the intelligent precautions taken by individuals.

### Wants and Workers.

[The attention of correspondents is directed to the fact that "Helps in Sickness and to Health" (Scientific Press 428, Strand) will enable them promptly to find the most suitable accommodation for difficult or special cases.]

Will any Hospital reader kindly inform an Irish nurse as to a hospital or other institution, preferably in Ireland, where a comfortable permanent home could be found for an aged invalid lady?

